# Hemodynamic Responses to Word Forms in Japanese Infant‐Directed Vocabulary in 5‐ and 9‐Month‐Old Infants: Early Sensitivity to Prosodic Structure and Emergence of Prosodic Representations

**DOI:** 10.1111/desc.70214

**Published:** 2026-05-07

**Authors:** Yoritaka Akimoto, Miki Takahasi, Naoto Yamane, Rachel Ka‐Ying Tsui, Akiko Hayashi, Reiko Mazuka

**Affiliations:** ^1^ Information & Management Systems Engineering Nagaoka University of Technology Niigata Japan; ^2^ RIKEN Center for Brain Science Wako Japan; ^3^ Center for the Research and Support of Educational Practice Tokyo Gakugei University Koganei Japan; ^4^ Department of Psychology & Neuroscience Duke University Durham North Carolina USA

**Keywords:** developmental change, fNIRS, infant‐directed vocabulary, Japanese, language acquisition, prosodic structure

## Abstract

The prosodic characteristics of a native language greatly influence early language acquisition. Yet, Japanese mothers are known to use a specific prosodic structure in infant‐directed vocabulary (IDV)—specifically, three‐mora, two‐syllable words with a heavy‐light pattern—which, crucially, differs from the standard prosodic rhythm of adult vocabulary. This study used near‐infrared spectroscopy to examine hemodynamic responses to the Japanese IDV form in 5‐month‐old (*n* = 31) and 9‐month‐old (*n* = 34) Japanese infants, targeting the period before and during the emergence of this preference. The results revealed that oxygenated hemoglobin was greater for the IDV form than for the non‐IDV form in the left superior temporal gyrus (STG) for both age groups, consistent with the advantage of the IDV form observed in previous behavioral studies. Furthermore, this effect was localized to the left middle and left posterior STG in 5‐ and 9‐month‐old infants, respectively, highlighting early sensitivity to its prosodic structure followed by the emergence of phonological representation. This cortical shift, along with an observed trend toward adult‐like patterns, may suggest a broader transition from perceptually accessible IDV structures to the more diverse patterns of standard adult vocabulary. Although 5‐month‐old infants who have not yet exhibited a preference for the IDV form may not have developed specific phonological representations, their brains' ability to process its prosodic pattern could serve as a foundation for subsequent learning. These findings demonstrate that the specific structure of Japanese IDV acts as a foundational scaffold, guiding the transition from initial prosodic tuning to mature word‐level processing.

**Summary:**

- Early sensitivity to prosodic structure was observed in 5‐month‐old Japanese infants.
- Emerging phonological representation was observed in 9‐month‐old Japanese infants.
- Japanese infant‐directed vocabulary form serves as a prosodic template.

## 1 Introduction

The prosodic structure of speech, including rhythm, provides a fundamental framework for early language acquisition. While traditional hypotheses focused on discrete rhythm classes (e.g., stress‐ vs. syllable‐timed), recent evidence suggests that infants utilize a more complex, multi‐dimensional array of prosodic and psychoacoustic cues (Paillereau and Chládková 2024). In most cases, these cues are salient in infant‐directed speech (IDS), which is characterized by distinct prosodic and acoustic features compared to adult‐directed speech, and is often assumed to play a facilitative role in language acquisition (Golinkoff et al. 2015; Soderstrom 2007; Spinelli et al. 2017; Saint‐Georges et al. 2013). To date, however, how specific aspects of speech modification are related to infants’ acquisition is not fully understood.

Modification in IDS sometimes involves the use of specialized vocabulary known as infant‐directed vocabulary (IDV), characterized by particular phonological forms that frequently employ onomatopoeia, diminutives, and reduplication (Gervain and Werker 2008; Laing 2019; Mazuka et al. 2008; Ota et al. 2018). However, the lexical composition of IDV varies significantly across languages. Whereas IDV in many languages frequently consists of simplified or modified versions of adult words (e.g., “doggy” for “dog”), Japanese IDV frequently employs distinct lexical labels (e.g., “maN.ma” for “gohan” [food]). As discussed in Tomosada (2005), based on surveys from three specific sites, approximately 40%–50% of Japanese IDV items are specialized lexical forms rather than adult‐derived terms. Moreover, Japanese mothers use onomatopoeic or mimetic forms as primary labels in 52% of their object references, whereas this occurs in fewer than 1% of English IDS (Fernald and Morikawa 1993). While English IDS does use salient onomatopoeia, these forms typically function as stylized “sound effect words” or “embellishments” to conventional nouns, rather than serving as independent primary labels for objects (Laing et al. 2017). In contrast, standard nouns are systematically replaced with repetitive forms in Japanese, accounting for 65% of Japanese IDV items (Mazuka et al. 2008).

Interestingly, how a dominant IDV form is related to the phonological system of the target language can also differ cross‐linguistically. For example, in a stress‐timed language, such as English, a heavy‐light bisyllabic form, with a stress on the first syllable, is called a trochee, and it is a form that occurs most frequently in adult speech. IDV forms that occur frequently in English tend to take this pattern as well, for example, night‐night, tu.mmy. As English is a stress‐timed language, where trochaic form occurs most dominantly (Cutler and Carter 1987; Cutler and Noris 1988), IDVs with the trochaic pattern can play a facilitative role in infants’ language development, for example, in recognizing and segmenting words (Jusczyk et al. 1993; Jusczyk and Aslin 1995).

In contrast, frequently used IDVs in Japanese take a form that does not occur frequently in adult Japanese, nor is it consistent with the mora‐timed rhythm of Japanese. In adult Japanese, the most frequent words are three‐mora, three‐syllable words, for example, To.yo.ta., or four‐mora, four‐syllable words, for example, Hi.ro.shi.ma. Yet, the overwhelming majority of Japanese IDVs are either three‐mora, two‐syllable heavy‐light (HL) words with a pitch accent on the first mora, such as “ma'N.ma” (“gohan” [food]), or four‐mora, two‐syllable words such as “waN'waN” (“inu” [dog]). Many of the IDV forms often bear little phonological resemblance to the corresponding adult word forms, either phonetically or prosodically (Mazuka et al. 2008, 2017). Remarkably, while the prosodic form of Japanese IDVs is not a form that occurs frequently in adult Japanese, it functionally resembles the trochaic pattern found in English. Hayashi and Mazuka (2017) found that Japanese infants begin to show a preference for pseudowords with this HL prosodic form over those with alternative prosodic structures (e.g., light‐heavy (LH)) between 4–6 and 8–10 months of age, replicating the emergence of English‐learning infants’ preference for the trochaic word forms (Jusczyk et al. 1993). Furthermore, the HL forms were found to facilitate Japanese infants’ segmentation of words from continuous speech, but not when words were presented in other forms (Hayashi and Mazuka 2010).

To understand how such specific modifications in IDV support the early stages of language acquisition, it is essential to consider the brain mechanisms that allow infants to tune into the prosodic distributional regularities prevalent in their linguistic environment. Neuroimaging evidence suggests that the neural mechanisms for rhythm categorization are active from birth, although this ability is primarily limited to differentiating between distinct rhythmic classes (Ortiz‐Barajas et al. 2023). Specifically, delta and theta oscillations allow newborns to differentiate between distinct speech rhythms, such as English versus French or Spanish, but not between French and Spanish. In addition to these electrophysiological measures, near‐infrared spectroscopy (NIRS) captures hemodynamic changes over longer durations, reflecting the brain's processing of rhythmic sequences rather than discrete, word‐level prosodic features. Utilizing this method, Abboub et al. (2016) demonstrated that early linguistic experience already modulates hemodynamic responses to rhythmic sequences. Their study showed that French‐exposed monolingual and bilingual newborns exhibit distinct hemodynamic responses to trochaic or iambic (weak–strong stress pattern) sequences of pure tones, based on prosodic cues of duration, intensity, or pitch. The results revealed that French‐exposed monolingual newborns exhibited the greatest activation in prosodic grouping based on the duration cue, with trochaic stimuli evoking greater activation in the left temporo‐parietal areas than iambic stimuli. In contrast, bilingual newborns, who were also exposed to another language that uses pitch in prosody to a much greater extent than French, exhibited the greatest activation in prosodic grouping based on the pitch cue, with iambic stimuli eliciting greater activation in the left temporo‐parietal areas than trochaic stimuli. These results suggest that greater hemodynamic responses are elicited when prosodic grouping patterns conflict with the native language, compared to when they are consistent.

As these early cortical sensitivities are further refined through ongoing linguistic exposure, they manifest as behavioral preferences for the prosodic structures prevalent in the infant's linguistic environment. For example, infants learning English or German initially prefer trochees over iambs; this preference emerges between 6 and 9 months of age in English (Jusczyk et al. 1993) and between 4 and 6 months in German (Höhle et al. 2009). Such prevalent patterns also play a crucial role in word segmentation. While English infants could correctly segment trochaic but not iambic words at 7.5 months, they could correctly segment both word types at 10.5 months of age (Jusczyk et al. 1999). Similarly, 9‐month‐old English and Dutch infants could segment trochaic words from fluent speech in Dutch (Houston et al. 2000). Furthermore, Friederici et al. (2007) utilized electroencephalography with an oddball paradigm to investigate stress‐pattern discrimination in 4‐ to 5‐month‐old infants. Using a crossed design where both trochaic and iambic patterns served as standards and deviants, they found that German infants, whose native language is trochaic‐dominant, exhibited a positive mismatch response to the non‐dominant iambic deviant but not the trochaic deviant. In contrast, infants learning French or Spanish, which are syllable‐timed languages, do not exhibit a general preference for trochaic stress patterns (Höhle et al. 2009; Pons and Bosch 2010). For instance, 9‐month‐old Spanish infants displayed either a trochaic or iambic preference depending on the position of the heavy syllable (Pons and Bosch 2010). Consistently, Friederici et al. (2007) demonstrated that 4‐ to 5‐month‐old French infants, who are exposed to a language characterized by phrase‐final prominence rather than initial lexical stress, exhibited a positive mismatch response to trochaic deviants but not iambic deviants.

Prosodic sensitivity becomes more nuanced in languages with higher rhythmic flexibility, such as European Portuguese. Specifically, Lu et al. (2024) found that 5‐ to 7‐month‐old infants learning European Portuguese exhibited positive mismatch responses to both trochaic deviants compared to iambic standards, and iambic deviants compared to trochaic standards. This dual sensitivity is observed despite the processing advantage for iambic stress in adult speakers (Lu et al. 2018) and the iambic preference observed in infants (Frota et al. 2020). This may be attributed to the specific linguistic environment of European Portuguese, which is characterized by a high frequency of both patterns and a specific rhythmic flexibility, unlike the more strictly timed German or French. However, P3a and late discriminative negativity components, associated with rapid involuntary attention switching and higher‐order auditory rule extraction, respectively, were elicited only in the iambic deviant condition, suggesting a prioritized neural commitment to the iambic pattern. Altogether, these findings show that the development of prosodic sensitivity, both at the behavioral and neural levels, is not merely a matter of maturation but rather the result of learning the prosodic distributional regularities prevalent in the infant's language environment.

While such language‐specific attunement has primarily been studied in the context of the dominant prosodic patterns in stress‐ or syllable‐timed languages, the case of Japanese IDV is particularly intriguing. As discussed above, the prosodic form of Japanese IDV is more similar to a trochee in a stress‐timed language than the typical adult words. Yet the effects of the trochee‐like IDV on Japanese infants’ word segmentation observed in behavioral studies seem to be in par with that of English‐learning infants. Thus, it has been proposed that the HL form serves as a foundational prosodic template for word‐like representations during early language acquisition, despite the fact that these forms do not occur prominently in the adult Japanese (Hayashi and Mazuka 2017). This proposal is also consistent with the observation that a large proportion of early words spoken by Japanese children are in the HL form (Ota 2006) and aligns with the argument that this form is the default for new word formation in the Japanese phonological system (Kubozono 1997, 2002). Therefore, examining how Japanese infants’ brains process the prosodic form of IDV may shed light on how language‐specific and language‐general prosodic properties contribute to language acquisition.

The cortical processing of prosodic cues involves functional hemispheric specialization that may shift as these cues become linguistically relevant (Minagawa‐Kawai, Cristià, et al. 2011). While “suprasegmental” features like melodic contours typically involve the right hemisphere, “segmental” or lexical rhythmic structures (such as lexical accent and stress) are processed primarily within the left‐hemispheric language network. This network includes the superior temporal gyrus (STG), a region crucial for early speech perception and processing of lexical prosody (Minagawa‐Kawai, van der Lely, et al. 2011). Consistent with this framework, Sato et al. (2010a) found that STG activation in response to Japanese pitch accent shifts from bilateral in 4‐month‐old infants to left‐dominant in 10‐month‐olds. It should be noted that cortical activation levels do not always increase linearly with development (Issard and Gervain 2018). Rather, whether cortical responses are greater for one stimulus over another depends on a non‐linear interaction between the infant's level of encoding and the resulting processing demands. For example, infant‐directed speech elicits stronger brain responses in infants than adult‐directed speech does (Naoi et al. 2012; Saito et al. 2007; Zangl and Mills 2007). Wagner et al. (2011) also found that neonates and 7‐month‐old infants exhibited greater hemodynamic responses to syllable sequences with immediate repetitions (i.e., an ABB pattern) than to unstructured syllable sequences (i.e., an ABC pattern). In contrast, 9‐month‐old infants exhibited greater hemodynamic responses to unstructured syllable sequences than to sequences with immediate repetitions (Gervain et al. 2008, 2012).

Based on this background, the present study used NIRS to investigate hemodynamic responses to the prosodic features characterizing Japanese IDV, focusing on activity within the STG. Specifically, we examined responses to HL and LH pseudowords in 5‐ and 9‐month‐old Japanese infants, focusing on the period before and during the emergence of a preference for the HL prosodic form. We employed stimuli in an adult‐directed register to isolate the processing of rhythmic structures from the exaggerated melodic contours of IDS, which are known to elicit strong right‐hemisphere activation (Naoi et al. 2012). Given that 4‐ to 6‐month‐old infants do not yet exhibit a behavioral preference for the HL form (Hayashi and Mazuka 2017), 5‐month‐old infants may exhibit similar hemodynamic responses to HL and LH forms. In contrast, differences in hemodynamic responses are expected in 9‐month‐old infants, as a preference for the HL form and the ability to segment it emerge at this age. However, differences in hemodynamic responses to HL and LH forms may already be present in 5‐month‐old infants or remain absent in 9‐month‐old infants, as brain activation and behavioral preferences do not always align (Sato et al. 2010a). Any differences between the HL and LH forms were expected to emerge in the left hemisphere, following the predicted left‐hemispheric dominance in linguistic processing and the use of an adult‐directed register.

## 2 Materials and Methods

### 2.1 Participants

Forty‐five full‐term 5‐month‐old infants (21 girls) and 55 full‐term 9‐month‐old infants (27 girls), all ethnically Japanese and acquiring Japanese as their native language, participated in a functional NIRS (fNIRS) session. Data from 14 5‐month‐old infants and 21 9‐month‐old infants were excluded from the analysis because they did not meet the criteria primarily owing to crying and refusing to wear probes, hair obstruction, and/or large motion artifacts. Consequently, the final sample consisted of 31 5‐month‐old infants (16 girls, mean age = 167.8 days, age range = 153–183 days) and 34 9‐month‐old infants (16 girls, mean age = 286.5 days, age range = 274–304 days).

All the parents provided written informed consent, and this study was approved by the ethics committees of [RIKEN] (Wako3 24‐11(13), March 23, 2015; Wako3 30–2(30), October 12, 2023). The study was conducted in Wako City, Saitama Prefecture, Japan. The sample was drawn from the general population in the Tokyo metropolitan area, which is an urban environment. In this study, individual‐level socioeconomic status data, such as parental income or education level, were not collected.^1^ However, the area where participants were recruited is generally characterized by a middle‐class socioeconomic profile, with high access to education and healthcare, based on publicly available demographic data.

### 2.2 Stimuli

**FIGURE 1:**
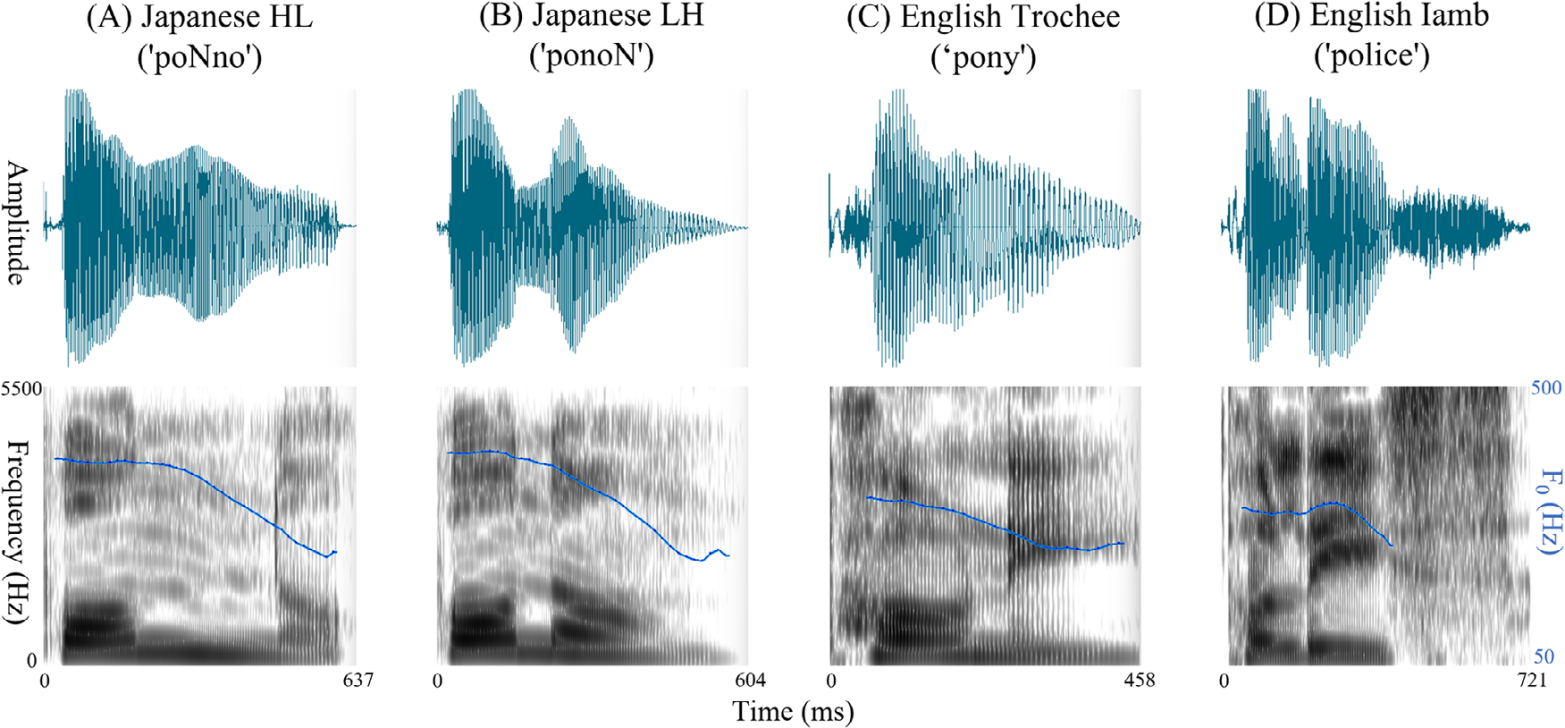
Spectrograms of the Japanese pseudoword stimuli (HL and LH forms). English bisyllabic words with trochaic (Strong–Weak) and iambic (Weak–Strong) patterns are also presented as a reference. Panels display the speech waveform (top) and spectrogram (bottom) for a representative example of each pattern: (A) a Japanese IDV pseudoword (“poNno”) with a three‐mora, two‐syllable HL structure and an initial pitch accent; (B) a Japanese three‐mora pseudoword (“ponoN”) with an LH sequence and an initial pitch accent; (C) an English two‐syllable trochaic word (“pony”) with strong–weak stress; and (D) an English two‐syllable iambic word (“police”) with weak–strong stress. English examples were visualized based on US‐style audio samples obtained from the Cambridge Dictionary (Cambridge University Press). Blue lines indicate fundamental frequency (F0) contours; HL, heavy‐light; LH, light‐heavy; ms, milliseconds.

To create the stimuli, 20 HL and 20 LH bisyllabic pseudowords spoken by a female native speaker of Japanese were recorded. Each LH stimulus was constructed using the same syllables as its corresponding HL form, but in reverse order (Figure 1). Half of the stimuli contained a long vowel, and the other half contained a moraic nasal. All items were spoken with an adult‐directed register and had a lexical pitch accent on the first syllable. The mean durations of the HL and LH stimuli were 687 ms (*SD* = 75) and 670 ms (*SD* = 69), respectively. These stimuli were selected from a pool of pseudowords used in a previous behavioral study (Hayashi and Mazuka 2017, Experiment 4). In this selection, the HL and LH items were specifically matched to ensure that the mean, maximum, and minimum values of stimulus duration, pitch, and intensity did not differ significantly between the two patterns.

### 2.3 Experimental Design

The experiment followed a block design with two conditions: HL and LH. For each condition, four distinct lists of stimuli were prepared, and one list was randomly selected for each block to prevent stimulus‐specific effects. Each 15‐s stimulus block consisted of one list containing 10 pseudowords, which were presented with a 1.5‐s stimulus‐onset asynchrony. Each infant was tested in a total of 14 blocks (7 HL and 7 LH blocks), with conditions presented in alternating order. Each stimulus block was preceded by a silent baseline period of either 18, 20, or 22 s, during which no stimuli were presented. The duration of the experiment was approximately 8–9 min, depending on the infant's state and the completion of all blocks.

### 2.4 NIRS Data Acquisition and Procedure

**FIGURE 2:**
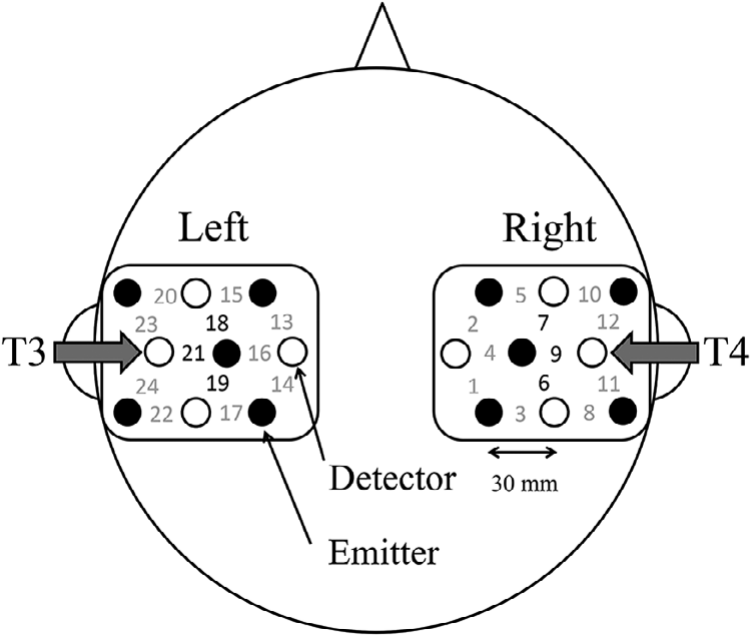
Placement of the near‐infrared spectroscopy probes. Black and white circles represent the emitter and detector, respectively. Measurement channels are numbered. The mid‐bottom detector probes for the left and right sides were placed near the T3 and T4 positions, respectively. The distance between the emitter and detector probes was 3 cm. Channels 6, 7, 9, 18, 19, and 21 are defined as regions of interest.

Hemodynamic responses were measured at a sampling rate of 10 Hz via a multichannel NIRS system (ETG‐4000, Hitachi Medical Corp., Tokyo, Japan) that used near‐infrared light with wavelengths of 695 and 830 nm. The placement of the NIRS probes followed previous studies (Minagawa‐Kawai, van der Lely, et al. 2011; Sato et al. 2010a; Yamane et al. 2021): five light‐emitting and four light‐detecting probes were attached to each lateral side of the head (Figure 2). The mid‐bottom detector probes for the left and right sides were placed near the T3 and T4 positions, respectively. The distance between the emission and detection probes was 3 cm. Eight emission and seven detection probes were also attached to the frontal region with the vertical midline and lowest line corresponding to the nasion–inion and Fp1‐Fp2 lines, respectively. However, these probes were excluded from the analysis because they often slipped out of place during the measurements.

Infants were seated on a parent's lap in a sound‐attenuated room. A loudspeaker (Reveal, TANNOY, Scotland, UK), located 70 cm from the infant's head, presented the stimuli at approximately 60 dB sound pressure level. The parent and one or two experimenters, who listened to music through headsets for masking, silently entertained the infant with toys. A silent movie was also played on the monitor in front of the infant. Each infant's movement was monitored and recorded by a ceiling‐mounted video camera. Approximately half of the infants participated in this experiment following the other NIRS experiment (i.e., a consonant discrimination task). However, infants exhibited similar response patterns, regardless of their participation in the other NIRS experiment. Consequently, they were analyzed together.

### 2.5 Analysis

fNIRS data were analyzed using the HOMER3 package version 1.80.2 (Huppert et al. 2009). Three right and left channel pairs, which presumably covered the superior temporal regions, were defined as the regions of interest (ROIs) (Minagawa‐Kawai, van der Lely, et al. 2011; Sato et al. 2010a; Yamane et al. 2021). After noisy channels with a low signal‐to‐noise ratio were excluded via “hmrR_PruneChannels,” with the parameter SNRthresh = 2, raw light intensity measurements were converted to changes in optical density via “hmrR_Intensity2OD.” Motion artifacts were identified using “hmrR_MotionArtifactByChannel,” with the parameters tMotion = 1.0, tMask = 1, AMPthresh = 4, and STDEVthresh = 15, and subsequently corrected by the spline interpolation using “hmrR_MotionCorrectSpline,” with the parameter *p* = 0.99. Wavelet filtering was also performed using “hmrR_MotionCorrectWavelet,” with the parameter IQR = 0.5. Residual artifacts were identified using “hmrR_MotionArtifactByChannel” with the same parameters, excluding blocks that contained residual artifacts. These parameters were selected according to Di Lorenzo et al.’s recommendations (Di Lorenzo et al. 2019). After band‐pass filtering between 0.01 and 0.09 Hz using “hmrBandpassFilt,” optical density data were converted to oxygenated hemoglobin (oxy‐Hb) and deoxygenated hemoglobin (deoxy‐Hb) concentration using “hmrOD2Conc,” with the parameter of the partial pathlength factor = 1. Finally, concentration data were averaged from −3 to 30 s after the onset of each stimulus block in each channel. Infants who had fewer than five available ROIs (where an available ROI was required to have three or more valid blocks in each condition) were excluded from the analysis.

To assess whether significant changes in oxy‐Hb or deoxy‐Hb occurred, the mean concentration change over 30 s was calculated from the onset of each stimulus block for each condition, channel, and age group. Channel‐wise one‐sample *t*‐tests against the baseline were conducted for each condition and age group. To control for multiple comparisons across the six channels, a permutation test with 10,000 repetitions was performed. For each permutation, the sign of the mean signal was randomly flipped for each participant, and channel‐wise *t*‐tests against the baseline were performed. For oxy‐Hb and deoxy‐Hb, the *t*‐value with the largest absolute value among the six channels was recorded to create a distribution of maximum absolute *t*‐values (i.e., a two‐tailed test). The observed *t*‐values from the original (non‐permuted) data were then compared against this distribution to determine significance. Furthermore, channel‐wise paired *t*‐tests were conducted within each age group to compare between the conditions. To control for multiple comparisons, a permutation test with 10,000 repetitions was performed. For each permutation, the condition labels were randomly flipped for each participant, and channel‐wise paired *t*‐tests between conditions were conducted. For oxy‐Hb and deoxy‐Hb, the *t*‐value with the largest absolute value among the six channels was recorded to generate a distribution of maximum absolute *t*‐values (i.e., a two‐tailed test). The observed *t*‐values from the original data were compared against this distribution to determine significance.

## 3 Results

**FIGURE 3:**
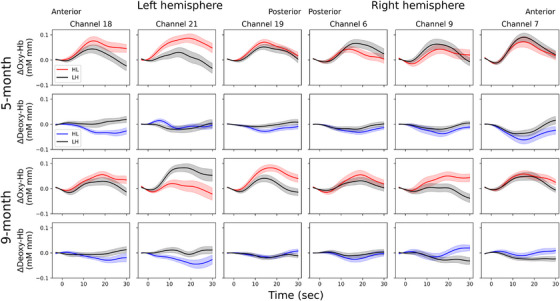
Time course of oxy‐Hb and deoxy‐Hb for heavy–light (HL) and light–heavy (LH) words in 5‐ and 9‐month‐old Japanese infants. Lightly shaded areas above and below the traces represent the standard error. Marks at 0 indicate the beginning of the stimulus presentation.

Figure 3 illustrates the time courses of oxy‐Hb and deoxy‐Hb responses for each condition in each age group. The results of one‐sample *t*‐tests against the baseline are presented in Table 1. Overall, bilateral responses were observed, although some differences were noted depending on age, condition, and Hb type. In 5‐month‐old infants, the HL condition demonstrated a significant increase in oxy‐Hb in left channels (CH18, 19, 21), while the LH condition exhibited significant increases in both left (CH19) and right (CH6, 7) channels. For 9‐month‐old infants, the HL condition displayed increased oxy‐Hb in the left (CH18, 19) and right (CH7) channels, and the LH condition showed an increase only in the left channel (CH21). Additionally, a significant decrease in deoxy‐Hb occurred in both the left (CH18) and right (CH6, 7) channels in the HL condition for 5‐month‐old infants, and in the right channel (CH7) in the LH condition for 9‐month‐old infants.

**TABLE 1:** The results of channel‐wise two‐tailed one‐sample *t*‐tests against the baseline for each condition and age group.

Hb	Age group	Condition	Hemisphere	CH	*t*	*p* (corr.)	*p* (uncorr.)	Cohen's *d*
Oxy‐Hb	5‐month	HL	Left	21	5.38	<0.001	<0.001	1.00
				19	5.09	<0.001	<0.001	0.91
				18	3.48	0.018	0.002	0.63
			Right	7	2.82	n.s.	0.009	0.51
				6	2.14	n.s.	0.041	0.38
		LH	Left	19	3.42	0.020	0.002	0.61
			Right	7	4.45	0.002	<0.001	0.81
				6	3.11	0.045	0.004	0.56
				9	2.50	n.s.	0.019	0.46
	9‐month	HL	Left	19	5.05	<0.001	<0.001	0.87
				18	3.14	0.041	0.004	0.54
			Right	7	3.87	0.006	0.001	0.69
				9	2.13	n.s.	0.041	0.38
		LH	Left	21	4.15	0.002	<0.001	0.72
			Right	7	2.76	n.s.	0.010	0.50
Deoxy‐Hb	5‐month	HL	Left	18	−3.70	0.008	0.001	0.66
				19	−2.67	n.s.	0.012	0.48
			Right	6	−3.34	0.022	0.002	0.60
				7	−3.11	0.041	0.004	0.57
				9	−2.46	n.s.	0.020	0.46
	9‐month	HL	Left	21	−2.60	n.s.	0.014	0.45
				18	−2.52	n.s.	0.017	0.43
		LH	Right	7	−3.27	0.022	0.003	0.59

*Note*: Negative *t*‐values indicate a decrease in Hb concentration relative to baseline. Corrected *p* values were derived from a permutation test with 10,000 repetitions.

Abbreviations: HL, heavy‐light; LH, light‐heavy; n.s., not statistically significant.

The results of the paired *t*‐tests between conditions are presented in Table 2. In both age groups, oxy‐Hb increased more in the HL condition than in the LH condition in the left channels (CH21 for 5‐month‐olds and CH19 for 9‐month‐olds). Additionally, 5‐month‐old infants exhibited higher deoxy‐Hb in the LH condition compared to the HL condition in the left channel (CH18), while 9‐month‐old infants exhibited higher oxy‐Hb in the LH condition compared to the HL condition in the left channel (CH21), although these differences were not significant after correction for multiple comparisons.

**TABLE 2:** The results of channel‐wise two‐tailed paired *t*‐tests between conditions for each age group.

Hb	Age group	Condition	Hemisphere	CH	*t*	*p* (corr.)	*p* (uncorr.)	Cohen's *d*
Oxy‐Hb	5‐month	HL > LH	Left	21	2.91	0.032	0.007	0.34
	9‐month	HL > LH	Left	19	2.81	0.037	0.008	0.30
		HL < LH	Left	21	−2.10	n.s.	0.044	0.30
Deoxy‐Hb	5‐month	HL < LH	Left	18	−2.15	n.s.	0.040	0.32

*Note*: Negative *t*‐values indicate a lower Hb concentration in the HL condition compared to the LH condition. Corrected *p* values were derived from a permutation test with 10,000 repetitions.

Abbreviations: HL, heavy‐light; LH, light‐heavy; n.s., not statistically significant.

## 4 Discussion

This study examined hemodynamic responses to HL and LH pseudowords in 5‐ and 9‐month‐old Japanese infants, corresponding to the periods before and during the emergence of a preference for the prosodic form of Japanese IDV. The results revealed that both HL and LH stimuli activated bilateral STG in both age groups at the uncorrected statistical threshold level, as evidenced by increased oxy‐Hb and decreased deoxy‐Hb compared to baseline. After applying multiple comparison correction, bilateral responses were still observed overall, with some differences depending on age, condition, and Hb type. However, a difference between HL and LH conditions was localized only to the left hemisphere channels even at the uncorrected statistical threshold level, consistent with the left‐hemispheric dominance for processing linguistically relevant rhythmic structures (Minagawa‐Kawai, Cristià, et al. 2011).

The comparison between conditions revealed that oxy‐Hb was significantly greater in the left channels for the HL condition than for the LH condition in both age groups. Additionally, although only at the uncorrected statistical threshold level, deoxy‐Hb was lower for the HL condition than for the LH condition in a left channel in 9‐month‐old infants. These results indicate that brain activation in the left STG were greater for HL than for LH word forms in Japanese infants of both age groups, aligning with previous behavioral studies demonstrating an HL advantage in Japanese infants (Hayashi and Mazuka 2010, 2017).

The 5‐month‐old infants showed greater activation for HL than for LH form words in the middle STG (CH21), while the 9‐month‐old infants showed this pattern in the posterior STG (CH19). This difference in response location likely reflects more advanced learning of HL form in 9‐month‐olds than in 5‐month‐olds. Although STG as a whole is believed to play an important role in speech processing, the middle STG has recently been shown to encode the acoustic onset edges (peakRate) of the speech envelope (Hamilton et al. 2021; Oganian and Chang 2019; Yi et al. 2019). This is not specific to linguistic stimuli, as the middle STG detects acoustic onset edges even for non‐speech tones, and the encoding of the speech envelope is independent of processing the complex spectral patterns that define consonants and vowels. However, the acoustic onset edges of the speech envelope clearly mark the onset of the syllabic nucleus, serving as cues to syllabic structure. Although the current study examines Japanese, which is based on a moraic rhythm, the detection of acoustic onset edges may also mark the time of the mora (Oganian and Chang 2019). Considering these points, the greater activation to HL compared to LH in the middle STG in 5‐month‐old Japanese infants suggests that the difference between the two conditions at this stage may be attributed to the detection of prosodic structure through the encoding of the speech envelope, with HL being processed more effectively in this way.

On the other hand, the posterior STG is well known as part of Wernicke's area, with its primary function considered to be phonological representation (Binder 2017; Buchsbaum et al. 2001; Wise et al. 2001). The posterior superior temporal regions disambiguate incoming auditory stimuli by comparing “phonological templates” formed through past experiences with new stimuli using a template‐matching algorithm (Warren et al. 2005). Therefore, the greater activation to HL compared to LH in the posterior STG of 9‐month‐old infants suggests that phonological representation is more advanced for HL than for LH in this age group. This interpretation is also consistent with the finding that Japanese infants acquire moraic rhythm relatively late, between 7.5 and 9.5 months of age (Sato et al. 2010b, 2012). Additionally, given the finding that 9‐month‐old—but not 7‐month‐old—infants can segment HL forms (Hayashi and Mazuka 2010), it seems that the detection of acoustic onset edges alone is insufficient for segmentation, and the development of phonological representations of prosodic structure in the posterior STG is necessary. This aligns with the findings that the posterior STG is not only involved in speech segmentation in both adults (Cunillera et al. 2009) and infants (Minagawa et al. 2017) but also sensitive to speech onset (Hamilton et al. 2021; Oganian and Chang 2019; Yi et al. 2019), which often marks boundaries between phrases or sentences.

This interpretation could also explain why 9‐month‐old infants showed greater oxy‐Hb in the LH condition than in the HL condition in the left middle STG channel (CH21) at the uncorrected statistical threshold. This may initially seem confusing, since greater responses to the HL compared to the LH were observed in the neighboring channel, with an increase in oxy‐Hb in CH19. This pattern may indicate that the processing of LH stimuli in 9‐month‐old infants is driven by the encoding of prosodic structure based on the speech envelope, similar to how HL stimuli are processed in 5‐month‐old infants, whereas the processing of HL stimuli in 9‐month‐old infants has progressed to the stage of phonological representation in the posterior STG. Furthermore, the opposite results in the same channel (CH21), with HL > LH in 5‐month‐old infants and HL < LH in 9‐month‐old infants, are consistent with the nonlinear relationship between learning stage and brain activation (Price and Devlin 2011; Taylor et al. 2013). This framework suggests that before a stimulus is learned, it elicits minimal brain activation because it is not yet fully processed by the brain. As learning progresses, brain activation increases, reflecting the greater processing effort required. When learning advances further, brain activation decreases as the stimulus becomes easier to process, requiring less cognitive effort. Therefore, the HL < LH pattern observed in CH21 at the uncorrected threshold may stem from individual differences, with some 9‐month‐old infants still exhibiting the HL > LH pattern characteristic of an earlier developmental stage.

Although preference and learning are distinct, in infants, the latter is typically accompanied by the acquisition of a preference for familiar stimuli, as well as a subsequent shift in the preference for novel stimuli (DePaolis et al. 2016; Wang et al. 2011; Houston‐Price and Nakai 2004). Infants initially prefer familiar to novel stimuli because these familiar stimuli have sufficient complexity in terms of learnability and learning outcomes. When learning is nearly complete, infants begin to prefer novel stimuli to familiar ones because further learning gains are not expected from highly predictable familiar stimuli. The timing of the familiarity‐to‐novelty shift depends on the complexity of the stimulus and the habituation time (Hunter and Ames 1988; Houston‐Price and Nakai 2004), which determines the quality of the resulting representation (Turk‐Browne et al. 2008). This mechanism enables infants to learn efficiently by allocating attention to information that is neither too simple nor too complex (Kidd et al. 2012). Familiarity preference and novelty preference are also associated with changes in brain activation (Nordt et al. 2016). When the same stimulus is repeatedly presented, brain activation can either increase or decrease during the familiarity preference phase and the novelty preference phase, respectively. Therefore, on the one hand, the increased brain activation to HL in the posterior STG in 9‐month‐old Japanese infants suggests that they are in the stage of developing representations of the Japanese IDV form, consistent with the results of a previous behavioral study indicating that 9‐month‐old Japanese infants showed a preference for Japanese IDV (Hayashi and Mazuka 2017). On the other hand, the finding that 5‐month‐old Japanese infants exhibited greater activation to HL than to LH in the middle STG suggests that infants were more sensitive to the prosodic structure of HL, particularly the encoding of acoustic onset edges based on the speech envelope, even before a preference for it emerged. This heightened sensitivity to HL may reflect early processing mechanisms related to detecting phonological features, such as the temporal structure of speech sounds. At this stage, although infants may not yet have developed specific phonological representations of the Japanese IDV form, their cortical ability to process the prosodic pattern of HL could serve as a foundation for subsequent language acquisition.

Furthermore, the observed trend toward increased sensitivity to the non‐IDV form in 9‐month‐olds may also correspond to a broader developmental transition in language development. The literature has indicated that as infants grow older, the emotional intensity of IDS decreases, with caregivers providing less exaggerated prosody to 9‐month‐olds than to younger infants in languages such as English (Kitamura and Burnham 2003) and Swedish (Schwarz et al. 2024). This corresponds to the period when infants’ preference for the highly exaggerated affective prosody of early IDS begins to diminish as they become increasingly sensitive to more complex adult‐directed speech patterns, suggesting a transition from affect‐driven to more informative, language‐focused processing (Newman and Hussain 2006; Kitamura and Lam 2009). In this context, this potential cortical shift might indicate that the brain's encoding targets are beginning to transition from familiar IDV structures toward the more diverse phonological patterns found in standard adult vocabulary.

In summary, this study provides evidence for a developmental progression in the hemodynamic processing of prosodic structure in Japanese infants, highlighting early sensitivity to the HL form in 5‐month‐olds and its phonological representation in 9‐month‐olds. These findings demonstrate that the specific structure of Japanese IDV acts as a foundational scaffold, guiding infants from initial prosodic tuning toward more mature word‐level processing. The present study advances our understanding of the brain mechanisms underlying language development, particularly in relation to the perception of prosodic structures that are fundamental to early language acquisition.

## Author Contributions

**Yoritaka Akimoto**: conceptualization, investigation, formal analysis, writing – original draft, writing – reviewing and editing, funding acquisition. **Miki Takahasi**: investigation, writing – reviewing and editing. **Naoto Yamane**: methodology, investigation, writing – reviewing and editing. **Rachel Ka‐Ying Tsui**: investigation, writing – reviewing and editing. **Akiko Hayashi**: conceptualization, resources. **Reiko Mazuka**: conceptualization, supervision, writing – reviewing and editing, funding acquisition.

## Funding

This work was supported by JSPS KAKENHI Grant Number 16K12451 (Yoritaka Akimoto) and 16H06319, 20H05617 (Reiko Mazuka), and by RIKEN Pioneering Research Project (Neurophysiological Mechanisms Toward Input‐Driven Language Development). These funding sources played no role in the research.

## Ethics Statement

This study was approved by the ethics committees of RIKEN (Wako3 24‐11(13), March 23, 2015; Wako3 30–2(30), October 12, 2023).

## Conflicts of Interest

The authors declare no conflicts of interest.

## Policy on Using ChatGPT and Similar AI Tools

During the preparation of this work, the authors used the free versions of OpenAI's ChatGPT and Google's Gemini to improve the readability of the manuscript's English expressions. After using these tools, the manuscript was further revised through English proofreading services. The authors thoroughly reviewed and edited the content as needed and take full responsibility for the content of the published article.

## Data Availability

Research data is available on the Open Science Framework (https://osf.io/ngcef/).

## Endnotes

1 It was the general policy of [RIKEN] that researchers were discouraged from collecting participants’ personal information unless it was directly related to the research question.
